# Reliability of the ICD-10 International Personality Disorder Examination (Urdu Translation): A preliminary study

**DOI:** 10.12669/pjms.306.5692

**Published:** 2014

**Authors:** Imran Ijaz Haider, Fatima Bukharie, Fareeha Hamid, Muhammad Ayub, Muhammad Irfan, Farooq Naeem

**Affiliations:** 1 Dr. Imran Ijaz Haider, MRCPsych*, *Professor of Psychiatry, Fatima Memorial Hospital, Lahore, Pakistan.; 2 Dr. Fatima Bukharie, Medical Officer, Fatima Memorial Hospital, Lahore, Pakistan.; 3 Fareeha Hamid, Clinical Psychologist, Fatima Memorial Hospital, Lahore, Pakistan.; 4 Dr. Muhammad Ayub, MRCPsych, Associate Professor of Psychiatry, Queens University, Kingston, Ontario, Canada.; 5 Dr. Muhammad Irfan, MCPS, FCPS, MS, Assistant Professor, Department of Psychiatry and Behavioural Sciences, Peshawar Medical College, Peshawar, Pakistan.; 6 Dr. Farooq Naeem, MSc, MRCPsych, PhD, Consultant Psychiatrist & Cognitive Behaviour Therapist, Associate Professor of Psychiatry, Queens University, Kingston, Ontario, Canada.

**Keywords:** International Classification of Disease (ICD), International personality disorder examination (IPDE), Urdu, Translation

## Abstract

***Objective:*** To test the reliability and applicability of Urdu translation of the International personality disorder examination (IPDE) in a psychiatric outpatient population in Pakistan.

***Methods:*** This study was conducted at the outpatient department of Fatima Memorial Hospital Lahore from April 2012 to March 2013. Patients considered to have a personality disorder by a psychiatrist were initially screened by the IPDE screening questionnaire. Those who scored positive on screening were evaluated in a detailed interview using IPDE. Two interviewers conducted the interviews simultaneously, to ensure inter-rater reliability. For translation, permission was taken from World Health Organization. Linguistic equivalence was assessed through back- translation and conceptual equivalence through opinion of mental health experts. The final Urdu draft was obtained after incorporating modifications suggested by experts following a feasibility study. The analysis was carried out using SPSS v.20.

***Results:*** Out of 30 enrolled patients, 25(83.3%) were females. The mean age of the sample was 28.5+6.08 years. Majority of patients had more than one personality disorder. Most prevalent personality disorder was emotionally unstable borderline type with a phi correlation of 0.831, followed by emotionally unstable impulsive type and anankastic personality disorder with phi correlations of 0.930 and 0.867, respectively, for definite cases. Correlation coefficient for dimensional scores between the two raters was 0.392 for paranoid personality disorder, 0.842 for anankastic and around 0.9 for the rest of the personality disorders, each.

***Conclusion:*** Urdu translation of IPDE is a reliable tool to screen and diagnose personality disorders in population of Pakistan.

## INTRODUCTION

The International personality disorder examination (IPDE) was developed within the Joint Program for the Diagnosis and Classification of Mental Disorders of the World Health Organization (WHO) and US Alcohol, Drug Abuse and Mental Health Administration (ADAMHA).^1^ The main aim was the development and standardization of diagnostic assessment instrument for use in clinical research around the world. Personality disorders are associated with a significant burden on the individuals with the disorder, those around them and on society in general but studies of the prevalence of personality disorders (PDs) have been fewer and on smaller-scale. The probability of consulting and receiving effective treatment from psychiatric services varies according to demography, degree of disability and diagnosis.^2^^,^^3^

The IPDE is a semi structured clinical interview, used as an evaluation tool to assess the personality disorders in ICD-10 (International classification of disease, 10^th^ edition) and DSM IV (Diagnostic and Statistical Manual of Mental Disorders, 4^th^ edition) classification systems.^1^ IPDE has two manuals to assess personality disorders, one according to DSM IV and the other according to ICD-10. Both these classificatory systems are overlapping but different.^4^^,^^5^ There is difference in nomenclature as well as in diagnostic criteria. ICD-10 has anankastic, anxious and dissocial instead of obsessive compulsive, avoidant and antisocial personality disorders respectively.^4^ Moreover in ICD-10 borderline and impulsive are considered subtypes of emotionally unstable personality disorder, and narcissistic personality disorder is not included.^4^ ICD-10 manual of IPDE consists of 67 items. It consists of a screening questionnaire as well, which consists of 59 items, which are self-reported by the patient as true or false.^1^

Overall, 14.79% of adult Americans (30.8 million), had at least 1 personality disorder. The most prevalent personality disorder in the general population was obsessive-compulsive personality disorder (7.88%), followed by paranoid personality disorder (4.41%) and antisocial personality disorder (3.63%).^6^ The un-weighted prevalence of personality disorders from a British survey showed that 10.7% of the sample (4.4% weighted) had at least one disorder, with men more likely to have a disorder (13.3%; weighted 5.4%) compared with women (8.7%; weighted 3.4%).^7^

Keeping in view that the basic assessment tool should be culturally applicable and reliable and easily understandable by local people, it was translated into different languages e.g. Greek, Hindi etc.^8^^,^^9^ The instrument is designed for use by clinicians (psychiatrists or clinical psychologists) experienced in the assessment of personality disorders.

As far as we are aware no study of prevalence of personality disorder was conducted in Pakistan. The aim of the current study was to test the reliability and the cultural applicability of the Urdu translation of the IPDE in Pakistan in a clinical outpatient population. The study follows WHO field test protocols and is focused on testing cultural appropriateness and inter-rater reliability.

## METHODS

This study was conducted at outpatient department of Fatima memorial hospital Lahore in a total duration of 11 months from April 2012 to March 2013. A purposive sampling method was used. Patients with clinical suspicion of personality disorders were screened by the screening questionnaire, and those who scored high on screening were evaluated in a detailed interview using IPDE (ICD-10 version). Patients with clinical evidence of organic brain disease, mental retardation or/and Psychotic illness were excluded from the study. The ethical clearance was given by Lahore Institute of Research and Development.

Patients considered to have a personality disorder using ICD10, RDC of possible personality disorder were referred by a psychiatrist (IIH). All these patients were independently screened by IPDE personality disorder screen. All the patients scoring above the cut off and were interviewed in detail. To ensure inter rater reliability two interviewers conducted the interview simultaneously, in which one of the interviewers performed the interview and the other was the silent observer who however had the ability to ask necessary questions at the end of interview for further clarification. Both interviewers scored patients independently and were not allowed to share the results during or after the interviews.

***International Personality Disorders Examination (IPDE)*** ^1^ ***: ***It consists of 157 items arranged under the following 6 headings: work, self, interpersonal relationships, affects, reality testing and impulse control. The items are introduced by open-ended inquiries and offer the individual the opportunity to discuss the topic and supplement the answers with examples or anecdotes. Additionally, the instrument provides a set of probes to determine whether the individual has met the frequency, duration and age of onset requirements. Scoring of items ranges between 0 (absent or within normal range), 1 (present to an attenuated degree) and 2 (pathological, meets criterion standards). The results include both a categorical diagnosis of personality disorders in both classification systems and a dimensional score for each personality disorder. Both can be obtained by paper and pencil algorithms, but also computer software is available.

***Translation*** ***& cultural adaptation: ***Permission was taken from the World Health Organization to translate the IPDE. Translation followed the strategy developed in our previous work.^10^ Translations were carried out by five bilingual mental health professionals. Linguistic equivalence was assessed through back- translation and conceptual equivalence through opinion of mental health experts. The final Urdu draft was obtained after incorporating modifications suggested by experts following a feasibility study on 5 outpatients. As recommended in the IPDE manual, two raters familiarised themselves with the ICD-10 module by interviewing 5 patients each with the Urdu draft. The IPDE was then administered by one of the two raters according to a preset randomized schedule. Interviewers received supervision by FN and IIH, throughout the project.

***Statistical Analysis: ***Analyses were carried out using SPSS version 20. The phi coefficient was used to test categorical diagnosis. This coefficient takes values from -1 (total disagreement) to +1 (total agreement). The value 0 means agreement just by chance. The Pearson Product Moment Correlation Coefficient was used to test the agreement between raters concerning the number of criteria met. Both coefficients take values from -1 (total disagreement) to +1 (total agreement). The value 0 means agreement just by chance.

## RESULTS

Out of 30 enrolled patients, 25(83.3%) were females and majority of the females (n=13) were housewives. The mean age of the sample was 28.5±6.08 years while the overall age range of the sample was 19 to 46 years with 19-38 years age range of females and 20-46 years age range for males. Eleven (36.7%) did not have any formal education. Most patients had more than one personality disorder (Table-I).

According to the analysis, most prevalent personality disorder was emotionally unstable borderline type. Rater one assessed 21 patients and rater 2 assessed 23 patients and they both agreed on diagnosis in 21 patients in total (phi correlation of 0.831 for definite cases). This was followed by emotionally unstable impulsive type (rater one assessed 18 patients and rater 2 assessed 19 patients and joint agreement was found in 18 patients) (phi correlation of 0.930 for definite cases) and anankastic personality disorder (rater one assessed 14 patients and rater 2 assessed 16 patients and joint agreement was found in 14 patients (phi correlation of 0.867 for definite cases) (Table-II).

Correlation coefficient was also calculated for dimensional scores between the two raters. It was 0.392 for paranoid personality disorder, 0.880 for schizoid, 0.890 dissocial, 0.956 for emotionally unstable impulsive, 0.938 for emotionally unstable borderline, 0.941 for histrionic, 0.842 for anankastic, 0.927 for anxious and 0.894 for dependent personality disorder.

## DISCUSSION

IPDE has been used extensively to detect various personality disorders.^11^^-^^14^ The results of this study suggest that the Urdu translation of the IPDE is a reliable tool to assess the local population with accuracy and can be easily used. The major problem with the IPDE is the time it takes to complete one assessment; however we found that IPDE screen can be used for initial assessment thus reducing the time.

The results are comparable with the related studies done for the same purpose, conducted in Greece and India.^8^^,^^9^ Thirty-one patients (12 male and 19 female) aged 35.25 ± 11.08 (range 19-54) years, took part in the Greek IPDE field trial^8^. Twenty-two non-psychotic outpatients (organic mental disorders, mental retardation and medical illnesses were excluded) in the age range of 18 to 60 years were assessed in Indian study done to check the reliability of IPDE ICD-10 module.^9^ In the Indian study, one third of the patients were suspected to have PD and two-thirds were women (n=15). Sample size and more females in above mentioned studies are comparable with our study. The Greek study reported that Antisocial and Borderline PDs were perfectly reliable with phi equal to 1.00.^8^ This is comparable to our study in which emotionally unstable borderline type was also found to have phi equal to 1. According to them, the diagnosis of any PD was highly reliable with phi >0.92 in both classification systems. On the contrary, not otherwise specified PD was not reliable at all with phi equal to 0. According to the results of Indian study, out of 22, 3 (13.6%) patients had definite and four patients (18.2%) had probable PD diagnosis.^9^ In contrast to that in our study all patients who were recruited after screening positive with the screening questionnaire were found to have a personality disorders. However, this is hardly surprising considering, only patients who were suspected to have a personality disorder were included in our study. They were further screened on IPDE screen and were found to be positive for a PD.

**Table-I:** Demographic details of the sample (n=30).

**Variable**		**Number**	**Percentage**
**Gender**	Male	5	16.7
	Female	25	83.3
**Occupation**	Student	6	20.0
	Housewives	13	43.4
	Employed	10	33.3
	Unemployed	1	3.3
**Age**	< 20	1	3.3
	20-29	16	53.4
	30-39	12	40
	40 +	1	3.3
**Marital Status**	Married	18	60
	Unmarried	12	40
**Educational Status**	No Formal education	11	36.7
	Primary	4	13.3
	Matriculation	4	13.3
	Higher Secondary	5	16.7
	Graduate	6	20

**Table-II:** Raters report and Phi Correlation Coefficients regarding specific personality disorders.

**Personality disorder (definite)** **Probable cases**	**Rater 1**	**Rater 2**	**Number agreed by both**	**Phi Correlation Coefficient**
Paranoid Probable cases	8 9	11 6	5 5	0.315 0.561
Schizoid Probable cases	14 5	14 7	12 4	0.732 0.586
Dissocial Probable cases	2 5	2 4	1 3	0.250 0.609
Emotionally unstable, impulsive Probable cases	18 0	19 1	18 0	0.930 -
Emotionally unstable, borderline Probable cases	21 5	23 5	21 5	0.831 1.000
Histrionic Probable cases	5 8	6 9	5 8	0.889 0.918
Anankastic Probable cases	14 4	16 5	14 4	0.867 0.870
Anxious Probable cases	13 5	13 5	12 4	0.864 0.760
Dependent Probable cases	12 4	13 4	11 4	0.795 1.000

Kappa value calculated when base rate more than 5%.

There are numerous limitations of this study. We had a rather small sample size. However, this is only a preliminary study and future studies will be planned in both community at risk populations (for example prisons etc) and psychiatric outpatient clinics with bigger samples and better methodology. Inclusions of only those who were suspected to have a personality disorder could be another source of bias. However, this study focused only on evaluation of psychometric properties of the instrument and it was not intended to be an epidemiological study. Although this translation was accomplished methods we developed in our previous work in which we translated ICD10, RDC, which included both translation and cultural adaptation of a large instrument, we did not use qualitative methods to further evaluate the effectiveness of this instruments. The feedback from the participants and the interviewers however, indicated that this translation of IPDE was acceptable and there were no major problems with comprehension and scoring of the items. The major issue however remains with generalization of these findings. The study was conducted in Lahore the second biggest city in Pakistan and the results might not be generalizable to patients from smaller cities or from the rural areas. Patients considered to have a personality disorder were referred by mental health professionals to the research team without the use of diagnostic criteria. However they were all found to have a personality disorder both on screening and detailed examination using IPDE.

Interviewers found the translated version to be easy to use and applicable in this study. Urdu translation of IPDE is therefore applicable in clinical practice and has sufficient inter-rater reliability. The major limitation of this study is a small size. There is a need to further this work in different populations and using better methodology.

## CONCLUSION

It is evident from the above mentioned results and discussion that the Urdu translation of IPDE is a reliable tool to screen and diagnose personality disorders in population of Pakistan.

## Authors’ Contributions:

**IIH **assessed patients for the inclusion in the study and supervised data collection.

**FB and FH **received training to conduct the study, led data collection and did data entry, and prepared the initial draft of the manuscript.

**MA **helped to revise the draft manuscript and gather references.

**MI **helped in data analysis and interpretation of results and preparation and revision of draft manuscript and supervised the research project.

**FN **conceived the idea and designed the study, supported other authors in drafting of the manuscript, performed statistical analysis and interpretation of results and overall supervised the study. All authors read and approved the final manuscript.
